# Winds of change: meteorological influences on Strokkur’s geyser eruptions, Iceland

**DOI:** 10.1038/s41598-025-26213-8

**Published:** 2025-11-04

**Authors:** Eva P. S. Eibl, Shaig Hamzaliyev, Guðrún Nína Petersen, Gylfi Páll Hersir

**Affiliations:** 1 Institute of Geosciences, University of Potsdam, Karl-Liebknecht-Str. 24–25, 14476 Potsdam, Germany; 2 Icelandic Meteorological Office, Bústadavegi 7–9, 105 Reykjavík, Iceland; 3 Independent researcher, Reykjavik, Iceland

**Keywords:** Seismology, Geophysics

## Abstract

Geothermal eruptions are among the most spectacular phenomena in nature. However, the regularity of the eruptions and the internal or external driving factors of eruptions remain unclear due to the scarcity of long-term data. Our study fills this gap by presenting an unprecedented 4.5-year catalog of 650,000 water fountains observed at Strokkur geyser, Iceland. Our findings reveal a correlation between the wind speed and the recurrence interval of eruptions. Specifically, as the wind speed increases, the recurrence interval increases quadratically. This represents the most significant external modulator, influencing the system at least down to the bubble reservoir at a depth of 24 m. A secondary modulator is the temperature, with a linear increase in the recurrence interval as the temperature decreases. A heat loss model that accounts for the wind speed and temperature differences between water and air does not fully predict the observed behavior. However, the model does not account for heat loss during fountaining or for water loss from the catchment during eruption. The aim of this study is to quantify the relationship between the weather and the geyser to enable a correction and to focus on other internal and external drivers in future studies.

## Introduction

Geysers are erupting hot springs that feature jetting water fountains. Geysers can either be driven by temperature instabilities in the conduit that lead to sudden flash-boiling of superheated water^1^ or by a steam bubble that accumulates in a reservoir and upon release, drives the fountain^2^.

Some geysers erupt regularly in the same style, while others have different eruption types. These eruptions are short and long eruptions^3–6^, minor and major eruptions^7^ or single to multi-tuple eruptions^8,9^. Geysers feature a delicate balance of a heat supply, a water supply and a conduit system that connects the subsurface reservoir to the surface. As such, they are affected by external factors such as the water supply^10^, the weather^11^, and landslides. Scientists strive to define the factors influencing these systems.

Geysers are divided into pool geysers, which have a pool on the surface, and cone geysers, which have a solid edifice on the surface. Reservoirs that feed cone geysers or geysers with deep cavities are not affected by variations in wind speed or air temperature^12^. Old Faithful is sufficiently insulated from surface conditions to have less variable eruption intervals^13^, and Steamboat’s intervals are not affected by wind speed, air pressure or seismicity^14^. Monitoring of thousands of geyser eruptions at El Tatio, Chile suggests that, while eruption intervals are independent of air temperature, wind, or barometric pressure, they may be subject to gradual variations from year to year^7,15^.

Pool geysers are affected by air speed, air temperature and evaporation^12,16,17^. The first reports of the effects of wind storms on the pool geyser Daisy, US, suggested that increased wind speeds lead to a lengthening of the eruption interval^18,19^. Further correlations were found at Geyser flat, Whakarewarewa, New Zealand^20^, Doublet Pool, US^17^ and Daisy, US^12^. However, with only 11 events in the latter study, no statistically reliable relationship can be established, and a thorough, long-term study is lacking.

Further geysers show changes in behaviour with respect to the air pressure or temperature. Shorter intervals were observed during high pressure^12,21^ but also during periods of decreasing barometric pressure^22,23^. Longer intervals were reported during periods of low air temperature^12^. Other studies reported a link between the geyser behaviour and air pressure or temperature without detailed discussion^24^. The hot spring Óþerrishola close to Strokkur, Iceland, in the Geysir area used to start erupting when the air pressure dropped just before it began to rain. Sigurður Greipsson, a farmer in the area, planned his work of drying and collecting grass according to Óþerrishola’s behavior (interview in 1965^25^). In the middle of the 20th century it erupted several times a day when it did not rain with a height around man’s height. Nowadays it does not erupt^25^.

Until now, establishing a reliable link has been difficult because of short eruption interval time series or a lack of nearby long-term weather records. There are currently few long-term datasets of geyser eruption frequencies^6,8^. We want to understand why geysers respond to the weather and to what depth the weather affects these systems. If we understand, quantify and account for the link between the geyser recurrence interval and the weather, we can reveal the remaining processes.

Strokkur is an ideal geyser for studying the long-term link between weather and eruption intervals because it erupts frequently, and high-resolution, long-term weather data exist nearby. We present an overview of our 4.5-year dataset and describe the correlation between weather and geyser eruption frequency and the effect on the deeper geyser system. We discuss the correlation between the wind speed and other weather parameters using a heat loss model and the eruption duration.

## Introduction to eruptions at Strokkur geyser

**Fig. 1:**
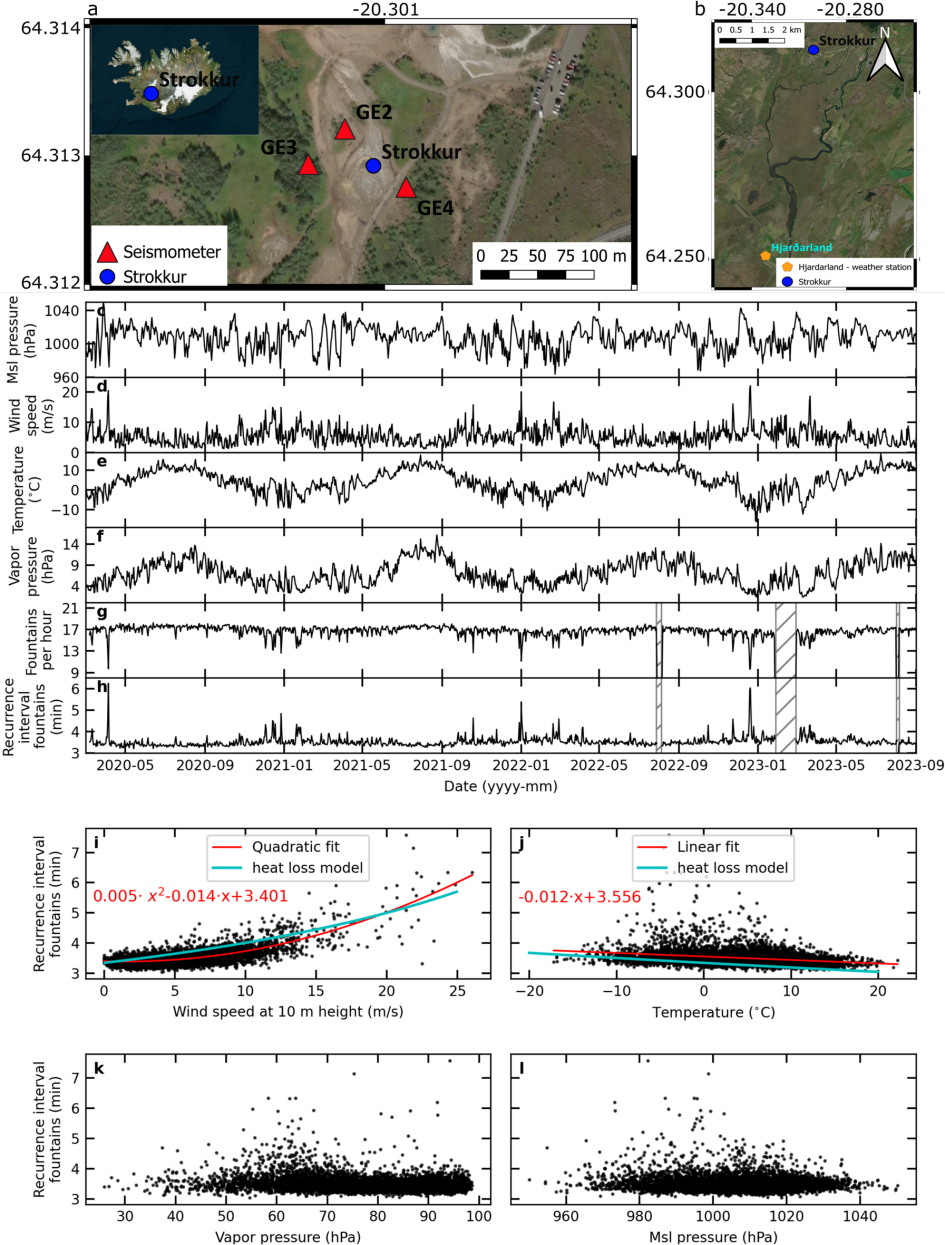
Wind speed affects the eruption frequency. (**a**) Geometry of the seismometer network around Strokkur, Iceland. The inset shows the location of Strokkur in Iceland. (**b**) Location of the weather station maintained by the Icelandic Meteorological Office at Hjarðarland. Map created using the Free and Open Source QGIS Desktop 3.36.3 (https://qgis.org/download/). One day average (**c**) msl pressure, (**d**) wind speed, (**e**) air temperature and (**f**) vapor pressure measured at Hjarðarland from March 2020 to September 2023. (**g**) Number of water fountains per hour and (**h**) mean recurrence interval of fountains both averaged per day. Hatched area indicates a data gap. (**i-l**) Correlation of mean recurrence interval in 6 h bins with (i) wind speed, (j) air temperature, (k) vapor pressure and (l) msl pressure. Best fit (red) and heat loss model (cyan) are shown.

The geothermally active region in Haukadalur in southern Iceland has been an attraction since the 18$^{th}$ century^26^. This active region is commonly called the Geysir area and hot springs cover an area of 3 km$^2$^27^, surrounding the geysers Strokkur and Great Geysir^28–30^ (Fig. 1). In the 19$^{th}$ century, Descloizeaux^31^ measured natural eruption intervals of 43 to 47 min and suggested that the variability of the temperature measurements in the Great Geysir’s pool may be influenced by differences in wind speed and direction, ground and air temperatures, and air humidity.

At that time, Descloizeaux^31^ described a water-filled basin at Great Geysir but no basin at Strokkur. Strokkur featured no water-filled basin according to observations in the 20th century where the water level was measured at 1 m depth in 1931^32^, at 11  m depth in 1937^33^, at 2 m depth in 1961^34^ and at 1.6 m in July 1963 when it was drilled to restart eruptions. Finally, the first report of a water-filled geyserite basin was in 1967 by Rinehart^35^ who reported about 12 m diameter. The water-filled pool was 58.7 m$^2$ in 2018^36^ and surrounds a central conduit of 2.2 m width^30^. At a depth of about 22 m in the conduit, a drillhole from 1963 was seen, extending to 39.4 m depth^30^.

The temperature at the surface in Strokkur varies a lot, depending on wind and surrounding temperature^37^. However, there are also variations in the pool water temperature throughout the eruptive cycle. In 2018 the water temperature in the pool increased to 82–87$^{\circ }$C about 15 s after the eruption^9^ and then decreased to 78–80$^{\circ }C$ about 1 to 2 min after the eruption.

Nowadays, 80$\%$ of Strokkur’s eruptions are single eruptions, followed by an average 3.7 min to the next eruption. Instead of single eruptions, **multi-tuple eruptions** with two to six water fountains occur at times, causing longer waiting times after eruptions of 6.2 to 16.4 min, respectively^8^. Within a multi-tuple eruption two to six water fountains occur at a temporal spacing of 16.1±4.8 s from each other before the eruption terminates and the recharge starts^8^. Each water fountain can consist of several water jets^38^. Fountains reach heights of 21.4±10.2 m^36^.

## Methods

### Seismic network

We used seismic network data from Strokkur from 27 June 2017 to 10 June 2018 (7L seismic network)^39^ and from 7 March 2020 to 30 September 2023. The sensors were installed at 38.8 m (GE4), 47.3 m (GE3) and 42.5 m (GE2) distance from Strokkurs’ conduit^8^ and data stored in data cubes and regularly downloaded (Fig. 1). In 2017/ 2018 we recorded data using broadband Trillium Compact Posthole 20 s seismometers (Nanometrics) sampling at 200 Hz. From 2020, we used Trillium Compact 120 s seismometers (Nanometrics) sampling at 200 Hz initially and at 100 Hz from April 2022.

### Creation of the water fountain catalog

We completed the 2017 to 2018 eruption catalog from Eibl^8^. We applied the Continuous Wavelet Transform^40^ to seismic data filtered from 9 to 25 Hz with an event detection threshold of 3 sigma to automatically detect geyser eruptions. The Continuous Wavelet Transform derives a Characteristic Function based on the density power time-frequency plane by a wavelet transform analysis^40^.

We then manually checked the detections and the time windows between them using the Pyrocko trace-viewer Snuffler^41^ on data filtered from 5 to 25 Hz. We removed false markers and added missing ones. Despite the previously reported anthropogenic noise and reduced SNR during the day^8^, we marked eruptions throughout the day using alternative filters of 3 to 9 Hz and 20 to 25 Hz. The final dataset comprises 144 690 water fountains in 2017/2018, including the 73 466 fountains reported by Eibl^42^ and statistically analysed by Eibl^8^. The same procedure was used to create an eruption catalog of 506 131 fountains between 2020 and 2023.

To remove any potential discrepancies between manually set markers by different people and automatically picked markers, we corrected the markers with respect to the emergent, seismic eruption onsets using an automatic method (Supplementary Fig. S1) as follows. We applied a bandpass filter between 3 and 11 Hz to all available components and then stacked the absolute values to combine all components into a single trace. We smoothed this sum trace by convolution with a Hanning taper window of length 2/3 s to obtain an amplitude envelope. We then separately applied two filters to this smooth envelope: A median filter of length 2 s to obtain a robust measure of transient signals of more than 2 s duration, and a 10$\%$-percentile filter of 20 s duration to measure the background noise level. The latter trace was further filtered with a 90$\%$-percentile filter of length 20 s, to compensate for the onset distortion introduced by the first filter. We divided the median filtered signal by the percentile filtered signal to obtain a smooth and robust signal-to-noise ratio, that is sensitive to transients of 2-20 s duration. To adjust the manual onset picks, we looked for where this ratio exceeded a threshold of 3. We only corrected the onset if such an automatic detection was within 2 s before or after the manually picked onset.

### Separation into eruption types and eruptive phases catalog

We separated the water fountains into single to sextuple eruptions (Supplementary Fig. S2 and Supplementary Fig. S3). As introduced by Eibl^8^, fountains with a temporal spacing of less than 46 s are considered as part of a multi-tuple eruption. Our waiting times and distribution of eruption types compare well with those published as an incomplete catalog^8^ and highlight the system’s stability and regularity from 2017 to 2023. Despite the regular eruption pattern in time, the mean waiting times after eruptions spread with standard deviations from 15.0$\%$ to 24.3$\%$ around the mean.

For these eruption types we assessed how the durations of the four phases in the eruptive cycle are affected by the weather from March to June 2020. We hence manually marked the eruption start and end, the start of the eruption coda, and the beginning of bubble collapses at depth in the conduit like Eibl^9^. We similarly mark the start and end of the seismic signal caused by the eruption, which is large in amplitude and broad in frequency. We mark the first seismic spike that is followed by several spikes at a temporal spacing of about 1.52±0.29 s in the so called eruption coda^9^. And finally when regular spikes at a temporal spacing of 24.5±5.9 s start, we mark the first one^9^.

### Weather measurements

To assess whether temporal changes between fountains correlate with changes of weather parameters, we used data from two weather stations. Firstly, we installed a Vaisala weather station on a hill 293 m northwest of Strokkur to measure the conditions at 1 m above the ground with 5 min sampling rate. It was powered with a 75 Ah battery that lasted only about a month. Hence, this setup was not feasible for a long-term comparison. Nevertheless, it confirmed that data from the automatic weather station Hjarðarland (88 m elevation), 7 km from Strokkur operated by the Icelandic Meteorological Office (IMO) (Fig. 1), could serve as a proxy for the weather conditions at Strokkur (Supplementary Fig. S4). The wind speed shows small scale differences caused by e.g. local topography. Station pressure and temperature correlate linearly while there is an systematic station pressure difference of 6 hPa due to the altitude difference of 60 m.

Hjarðarland weather station records surface pressure at 1.5 m a.g.l., air temperature and humidity at 2 m a.g.l. and wind speed and direction at 10 m a.g.l., at 10 minute interval. Other parameters, e.g. mean sea level pressure (mslp) and vapor pressure were calculated using WMO conventions^43^. Data obtained for this study are mslp, air temperature, relative humidity and vapour pressure as well as wind speed and direction for the periods of the seismic catalog, June 2017 to June 2018 and March 2020 to September 2023. The wind speed and direction are 10 minutes averages. Other measurements are 1 minute averages for the last minute. The vapor pressure is the pressure due to the vapour in the air and is thus a function of the specific air humidity while relative humidity is a function of both the specific humidity and temperature. Although wind is on a large scale a function of the horizontal pressure gradient force, wind speed and direction are also impacted by the orography and have quite fast temporal and spatial variations.

### Comparison of weather data and eruption catalog

To compare 4.5 years of weather and eruption data, we calculated the mean recurrence interval of fountains independent of eruption type. After two consecutive single eruptions, the average waiting time to the next eruption is 3.7 min. An average double eruption has a short interval of 14.2 s and a longer one of 6.2 min (Supplementary Fig. S2d, e and t and Supplementary Fig. S3d, e and t), yielding an average mean recurrence interval of fountains of 3.2 min. The recurrence intervals reported here are thus slightly smaller than the waiting times after single eruptions reported by Eibl^8^.

Changes in the mean recurrence interval of fountains are not biased by the haphazard temporal sequence of single and multi-tuple eruptions, as we repeated our analysis using only single eruptions. This did not change the correlation presented in the Results section of this paper.

We investigated the seismic data and weather data using the Python toolbox Obspy^44^. We binned the recurrence intervals between fountains, the duration of the four phases in the eruptive cycle for single to quadruple eruptions, number of eruptions and weather parameters such as wind speed, air temperature, msl pressure and vapor pressure. We investigated bins from 1 h to 24 h and correlated the weather parameters with the geyser parameters over the course of 4.5 years. The correlation increased and converged for increasing window lengths (Supplementary Fig. S5) since the eruption types are not distributed evenly in time and this effect is smoothed in longer time bins. We chose 6 h long time windows since it averages the uneven distribution of eruption types and at the same time still allows to resolve short-term changes in the weather parameters.

## Results

### The weather conditions at Strokkur

The Icelandic climate is generally maritime with cool summers and mild winters. For the 10 year period 2010–2019 the mean annual temperature at Hjarðarland was 4.4 $^{\circ }C$. December is the coldest month of the year at -1.5 $^{\circ }C$, and July the warmest at 10.9 $^{\circ }C$. During winter the temperature varies little or not diurnally. The windiest month is February and the calmest July. The most common wind directions are northeasterlies, followed by southsouthwesterlies, especially during summer due to sea breeze (Supplementary Fig. S6).

The weather conditions during the time period March 2020 to September 2023 are shown in Fig. 1 and Supplementary Fig. S7. The mean sea level pressure varies from 960.3 hPa on 21 September 2021 to 1050.5 hPa on 28 March 2020, with a mean value of 1005 hPa (Fig. 1c).

Wind speeds above 12 m/s occur only when the air temperature ranges from -6 to +7 $^{\circ }C$. This is due to the maritime climate, as periods with low winter/ warm summer temperatures are generally associated with low wind condition and little mixing. The mean wind speed during the period is 5.4 m/s, similar to the climatic values, calculated for 2010-2019^45^. Wind speed ranges from 0 m/s to 28.7 m/s (Fig. 1d). The three highest values 28.7 m/s on 1 January 2022, 28.6 m/s on 28 February 2022 and 28.4 m/s on 5 April 2020 are at or near days of low number of fountains per hour.

The mean temperature is 4.4 $^{\circ }C$ (Fig. 1e), in line with the climatic value, with a maximum of 26.5 $^{\circ }C$ on 9 July 2023 and minimum of -19.3 $^{\circ }C$ on 1 January 2023.

The vapor pressure has a mean value of 7.1 hPa (Fig. 1f), varying between 1.1 and 19.6 hPa with generally higher values during summer and lower during winter.

### Overview of eruption frequency at Strokkur

In the time span analysed here, Strokkur features 16.4±3.1 fountains per hour (Fig. 1g and Supplementary Fig. S7e). While increases in fountain number to more than one standard deviation usually last one hour, decreases in fountain number by more than one standard deviation persist several hours. Notable deviations occur on 21 November 2017 when Strokkur only features 11 fountains per hour, and on 5 April 2020 and 1 January 2022 when it features only 7 fountains.

The mean recurrence interval is 3.5±0.4 min (Fig. 1h). Within our 4.5 year catalog it ranges from 2.3 to 9 min. While the 11 fountains per hour in November 2017 coincide with a recurrence interval of 5.3 min, the 7 fountains in 2020 and 2022 increase the recurrence interval to 8.7 and 9 min, respectively.

### Higher wind speed reduces the eruption frequency of Strokkur

The recurrence interval of fountains correlates strongly with wind speed (Fig. 1i). Calm conditions coincide with a recurrence interval of 3.4 min and 17 eruptions per hour. Wind speeds of 24 m/s coincide with recurrence intervals of 6.7 min and 8 eruptions per hour. The wind speed causes the recurrence interval to increase following a $x^2$ trend. The correlation is reliable up to 15 m/s and is present throughout the year. The wind speed has an immediate effect on the geyser (Supplementary Fig. S8a and b, f and g, k and l).

For an air temperature increase from -12 to 20$^{\circ }C$, the recurrence interval linearly decreases from 3.7 to 3.3 min (Fig. 1j). The vapor pressure and msl pressure do not correlate with the recurrence interval (Fig. 1k and l).

### Wind speed affects the geyser system down to the bubble trap

**Fig. 2:**
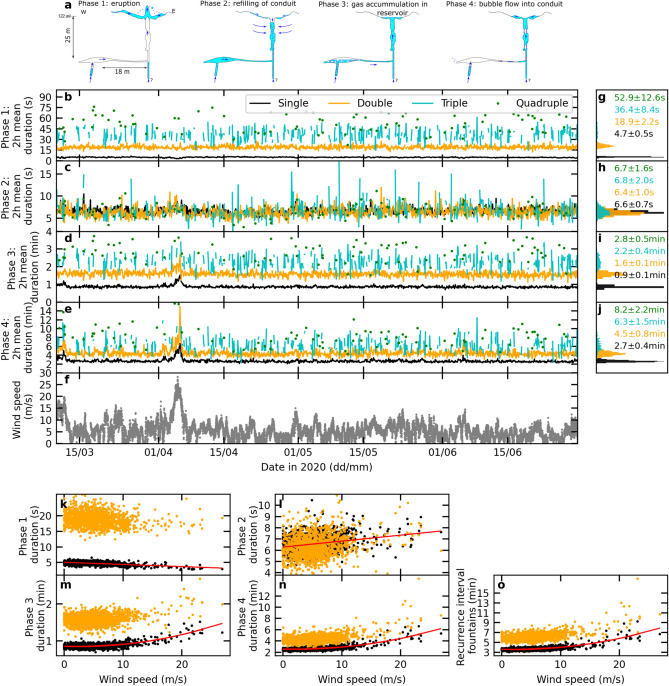
The wind affects the system down to the bubble reservoir. (**a**) Overview of phases in the eruptive cycle modified from Eibl^9^. (**b-e**) Mean duration in 2 hour bin of (**b**) Phase 1, (**c**) Phase 2, (**d**) Phase 3 and (**e**) Phase 4 in comparison to (**f**) wind speed. Colors highlight averages for single (black), double (orange), triple (cyan) and quadruple (green) eruptions. (**g-j**) Histograms and averages of subfigures (**b-e**). (**k-o**) Mean duration in 6 hour bin of (**k**) Phase 1, (**l**) Phase 2, (**m**) Phase 3, (**n**) Phase 4 and (**o**) the whole eruptive cycle from the start of one eruption to the start of the next one shown for single and double eruptions. The (**k, l**) linear and (**m, n, o**) quadratic polynomial fits the mean phase duration for single eruption (red).

The geyser passes from one eruption to the next one through an eruptive cycle with four phases that are steady over time^9^. Phase 1 features one or multiple water fountains on the surface (Fig. 2a). Most of these water fountains start with the formation of a water bulge before the bulge bursts and water jets into a fountain^36^. In Phase 2 the conduit refills with water while the seismic amplitude is low. Phase 3 features a seismic eruption coda generated by processes in the bubble reservoir. Finally, in Phase 4 bubbles leave the bubble reservoir, move into the conduit and collapse at depth at a regular temporal spacing shortening from 27 to 23 s within Phase 4. While Phase 2 is of similar duration for different eruption types, the duration of Phases 1, 3 and 4 increases from single to quadruple eruptions. Further details on the duration of the phases for different eruption types can be found in figure 5 and Table S1 in Eibl^9^ and Fig. 2g-j.

Here we separately assess how the wind speed (Fig. 2f) affects these phases (Fig. 2b to e). For single eruptions the duration of Phase 1 linearly decreases from 5.4 to 3.2 s with wind speeds increasing from 0 to 23 m/s (Fig. 2k). For the same wind speed range, the duration of Phase 2 and hence the time it takes to refill the conduit increases slightly (Fig. 2l). Phase 3 and Phase 4 (Fig. 2m and n) both follow the $x^2$ trend that is visible when assessing the whole eruptive cycle (Fig. 2o). They increase from 0.85 to 1.3 min and 2.6 to 5.1 min, respectively.

## Discussion

We find a strong relationship between the recurrence interval of fountains and the wind speed. The geyser reacts immediately to an increase in wind speed. Similar correlations have been documented at Daisy, US^12,18,19^ and Geyser flat, Whakarewarewa, New Zealand^20^. Hurwitz^12^ found that pool geysers are sensitive to wind speed and temperature, while we only find a strong correlation with wind speed. A study of 11 wind storms^12^ found that at least one eruption interval at Daisy lengthened by more than 1 standard deviation above the monthly mean during 8 of 11 storms with peak wind speeds of more than 7 m/s. However, for two wind storms no change in intervals was observed and for one the interval was shorter than the average minus one standard deviation. For two further measurements of lengthened intervals, faulty instruments were suspected to have lead to erroneously large values^12^. We here report a clear increase in interval with increased wind speeds.

At Geyser flat, Whakarewarewa, New Zealand^20^ increased wind speed in certain directions led to a drop in the water level at Te Horu and higher activity at Pohutu. In certain wind directions water was blown from the catchment and the recharge of water reduced. At Steamboat less volume discharged at higher wind speeds (4 instead of 1 m/s) indicating that water was diverted from the usual drainage channels^14^. Here, we do not observe a change in water level, but due to the slope and predominant wind directions from the NE (Supplementary Fig. S6) we hypothesise that water is lost from the catchment during high wind speeds.

Hurwitz^12^ reported shorter eruptions in cold air conditions and longer recurrence intervals during large wind speeds and deduced heat loss and evaporation as major influencing factor. Marler^18^ also inferred more water cooling and evaporation in strong wind. Our data suggest that strong wind cools the water in the pool leading to unfavorable conditions for eruptions and longer intervals between eruptions. Higher wind speed should cause more efficient cooling of the ejected water by transporting away heated air and water vapor from the surface of the hot water. The enhanced temperature gradient created in this way causes a higher heat flow and the loss will be especially high at low ambient temperatures. The fountain water that flows back and other inflowing meteoric water from the surface and sides, will be colder than during lower wind speed. Eibl^9^ observed that the pool temperatures plateau before an eruption and increase shortly after an eruption. At that stage the pool might be filled with fluids that were much deeper in the system prior to the eruption, and thus are intrinsically hotter. The subsequent decrease of temperature is due to cooling of the pool via heat transfer to the atmosphere.

We compared our observed relationship with a heat loss model applied in Hurwitz^12^. An amount of energy *U* needs to be added to the water volume in the pool after eruption to allow an eruption. The rate at which a deep heat source provides this energy is *H* and with time heat *E* per unit area is lost depending on the pool area *A*:

1 $$\begin{aligned} U = (H - A \cdot E) \tau \end{aligned}$$

Here, $\tau$ is the recurrence interval. The eruption lasts a few seconds while the mean recurrence interval is 3.7 min. The heat loss per unit area is estimated as^46^:

2 $$\begin{aligned} \sqrt{(2.7 \cdot (T_p-T_a)^{(1/3)})^2 + (8.1 \cdot A^{-0.05} \cdot W_s)^2} \cdot e_s - e_a \end{aligned}$$

where $W_s$ is the wind speed in m/s, *T_*_p_ the pool temperature and *T_*_a_ the air temperature. Similar to Hurwitz^12^ we assume the difference of the near-surface vapor pressure *e_*_s_ and ambient vapor pressure *e_*_a_ is approximately the near-surface vapor pressure.

We first use $\tau =3.4$ min at 1 m/s wind speed and $\tau =4.55$ min at 16 m/s wind speed to calibrate the model. We extrapolate the wind values from 10 m to 1 m height according to the log wind profile^47^

3 $$\begin{aligned} W_s(z_2) = \frac{W_s(z_1)}{ \frac{log((z_2-d)/z_0)}{log((z_1-d)/z_0)}} \end{aligned}$$

where $z_2$ is 1 m height, $z_1$ is 10 m height and *Ws* the mean wind speed at the respective height. We assume a roughness length $z_0$ of 0.03 m for the roughness of open terrain (grassland) on wind flow and a zero-plane displacement *d* of 0 m where we assume the mean wind speed is zero^47^.

According to the calibrated heat loss model, we calculate *H* and *U* for Strokkur. A net energy input *U* of 283 kJ is needed to start an eruption and the deep inflow energy rate *H* is 132 kJ/min. The model however, underestimates the true heat loss and predicts smaller recurrence intervals than we observe at high wind speeds. This model accounts for free and wind-forced convection in a large pool. It does not account for hot water loss from the catchment or additional cooling while water is jetting into the air. At high wind speeds we could envisage the water to be distributed over a larger area, increasing *A* and hence the cooling area. So *A* could be dependent on the wind speed. In addition, the temperature difference of the fountain water to ambient air might be larger than for the pool and ambient air, increasing the cooling. This discrepancy might be larger for erupting hot springs causing a larger increase in the recurrence interval than predicted. Doublet pool in contrast has a pool surface of 10 m$^2$ where bubbles collapse without water ejection into the air^17^.

It is noteworthy that the longer recurrence interval at higher wind speeds is due to a longer Phase 3 and 4. In Phase 3 the main activity focuses in the bubble trap at 23.7±4.4 m depth^9^. Strong winds hence affect the water in the bubble trap. As the area where air touches the water surface is just 58.7 m$^2$^36^ and most of the water surface is in contact with insulating rock, we speculate that either colder water from the surface recharges the bubble trap or colder water from the conduit is sucked into the bubble trap when the eruption ends.

A reduced msl pressure could impact the periodicity by reducing the boiling temperature and increasing the number of fountains per hour. However, here we observe a decrease in fountain number per hour. This change in boiling temperature at Strokkur is too small to have a measurable effect and the other effects outweigh it.

Despite strong changes in pressure for example in April 2020, they have no effect on Strokkur (Supplementary Fig. S8). While a pressure drop on the conduit e.g. due to a change in water column level should lead to more eruptions, the whole connected system experiences a pressure drop and hence does not react.

Influences of barometric pressure and air temperature were reported at other geysers worldwide. Daisys’ eruption intervals showed a 1 cycle per day peak similar to the barometric pressure and air temperature^12^, where air temperature had a stronger effect than the barometric pressure and intervals were longer during low air temperatures. Intervals were longer during low barometric pressure at Daisy, US^12^, Old Faithful, US^21^ and the geyser in Atami, Japan^23^. Since the temperature of some hot springs also increased during low pressure, Honda^23^ argued that low pressure caused more flow and circulation of water that might then prolong the heating process in a cavity. In contrast, Splendid geyser in the 1950s had 90$\%$ of eruptions during storms or times of falling barometric pressure^22^. For other springs in that area such as Old Faithful, no effect could be found. Rojstaczer^24^ found an effect of atmospheric pressure changes of more than 5 hPa influenced the eruption frequency of Big Anemone, Plume, Riverside, Yellowstone.

We observe slightly fewer eruptions during lower air temperatures. Similarly, Steamboat had more eruptions in summer^14^. In contrast, Liu^17^ reported a weak correlation between fewer eruptions and large temperatures and argued that during daytime wind speeds were also higher. Finally, precipitation might also play a role as Hurwitz^6^ found more frequent eruptions in Yellowstone in years with high precipitation.

Finally, the seismic signal associated with eruptions is shorter in duration during high wind speeds. However, eruptions are likely not shorter during high wind speeds. Since the bulge formation does not cause seismic signals^36^, we merely record the bursting of the bulge, the explosion and water splashing on the ground. Thus the seismic duration seems shorter due to the increased wind noise. The eruption signal will then emerge later from the noise but be hidden earlier. In addition, the seismic impact of the erupted water might be reduced when high wind speeds transport the water from the catchment.

We conclude that pool geyser Strokkur in southern Iceland looses heat to the atmosphere from a water-filled pool on the surface. This escalates with increasing wind speed as the surface area and cooling of the water fountain increase. In addition, hot water from the fountain is lost from the catchment. Strong winds affect the system at least down to the bubble reservoir at 23.7±4.4 m depth since only the phases of the eruptive cycle persist longer that as associated with recharge processes at depth. We establish a reliable relationship between the wind speed and the recurrence interval of fountains. This enables us in the future to correct for it and study further factors influencing this delicate system in more detail.

## Supplementary Information

Supplementary Information.

## Data Availability

The 2017/18 seismic data and eruption catalog are available at GEOFON^39^ and GFZ Data Services^42^, respectively. The 2020 to 2023 seismic data are available at^48^ and the catalogs are available at^49^. The weather data are available from the Icelandic Meteorological Office. The point of contact for any data or questions regarding the study is Eva P. S. Eibl.
