# Supplementary material for: Winds of change: meteorological influences on Strokkur’s geyser eruptions, Iceland
Source: Sci Rep. 2025 Nov 4;15:38462. doi: 10.1038/s41598-025-26213-8 (PMC12586530; doi:10.1038/s41598-025-26213-8)
Supplement: Supplementary file 1 — Supplementary Information. [file 41598_2025_26213_MOESM1_ESM.pdf]

Supplementary Information of "Winds of Change: Meteorological Influences on Strokkur's Geyser Eruptions, Iceland"

Eva P. S. Eibl, Shaig Hamzaliyev, Guðrún Nína Petersen, Gylfi Páll Hersir

Content: Supplementary figures S1-S8

References

1. Eibl, E. P. S. *et al.* Eruption interval monitoring at Strokkur geyser, Iceland. *Geophys. Res. Lett.* **47**, DOI: 10.1029/2019gl085266 (2020).
2. Holmes, J. Wind Loading of Structures (2nd ed.). *CRC Press.* DOI: 10.4324/9780203964286 (2007).

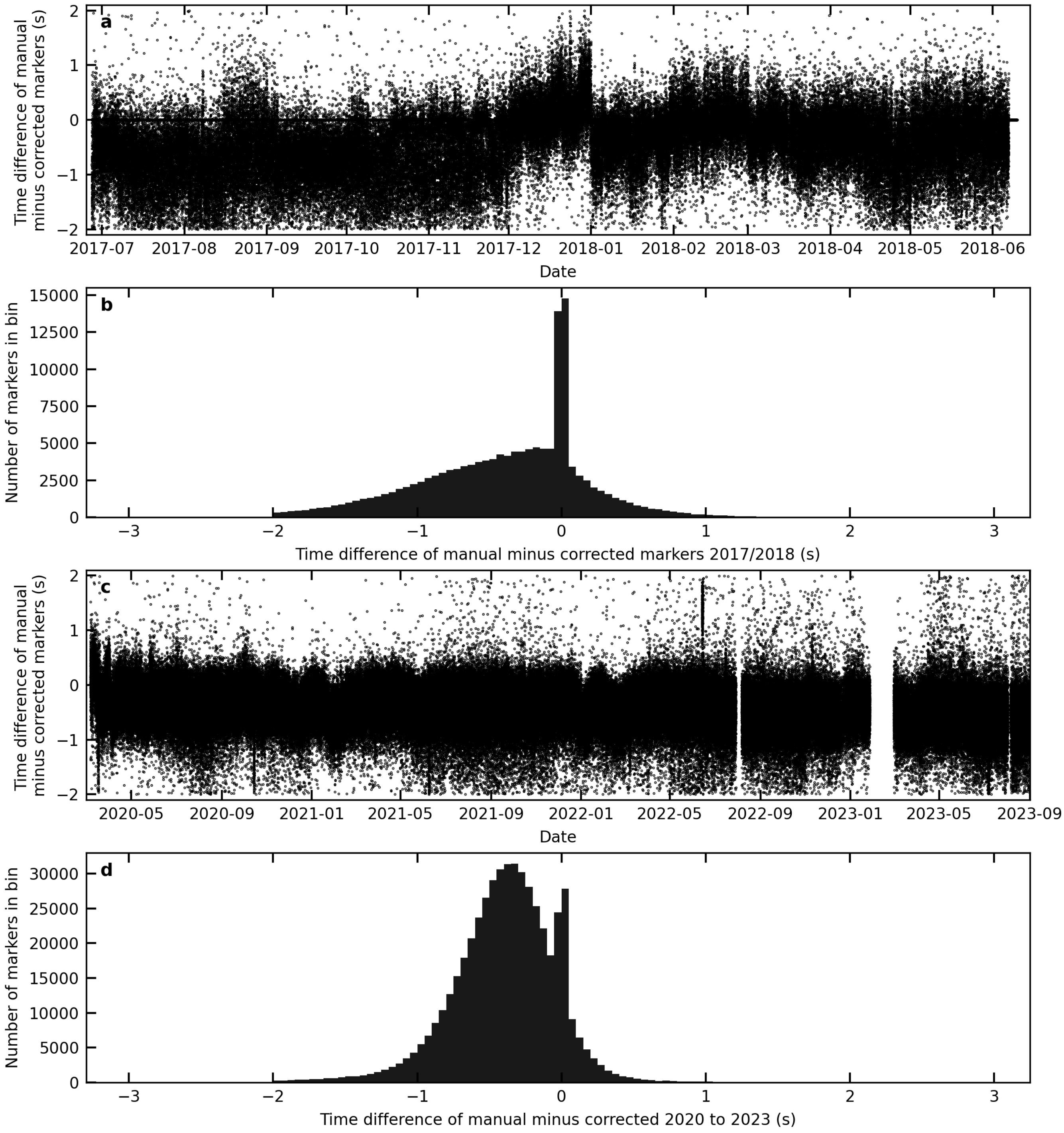

Figure S1. Improved geyser markers using an automatic approach of a short and long time window. (a) The temporal pattern in marker difference shows different manual marking patterns in 2017 to 2018. (b) The distribution of the applied time correction is skewed. (c) The markers from 2020 to 2023 were mostly set using CWT. (d) The correction of markers from 2020 to 2023 is Gauss distributed i.e. the CWT approach marks eruptions a bit later than our automated correction approach.

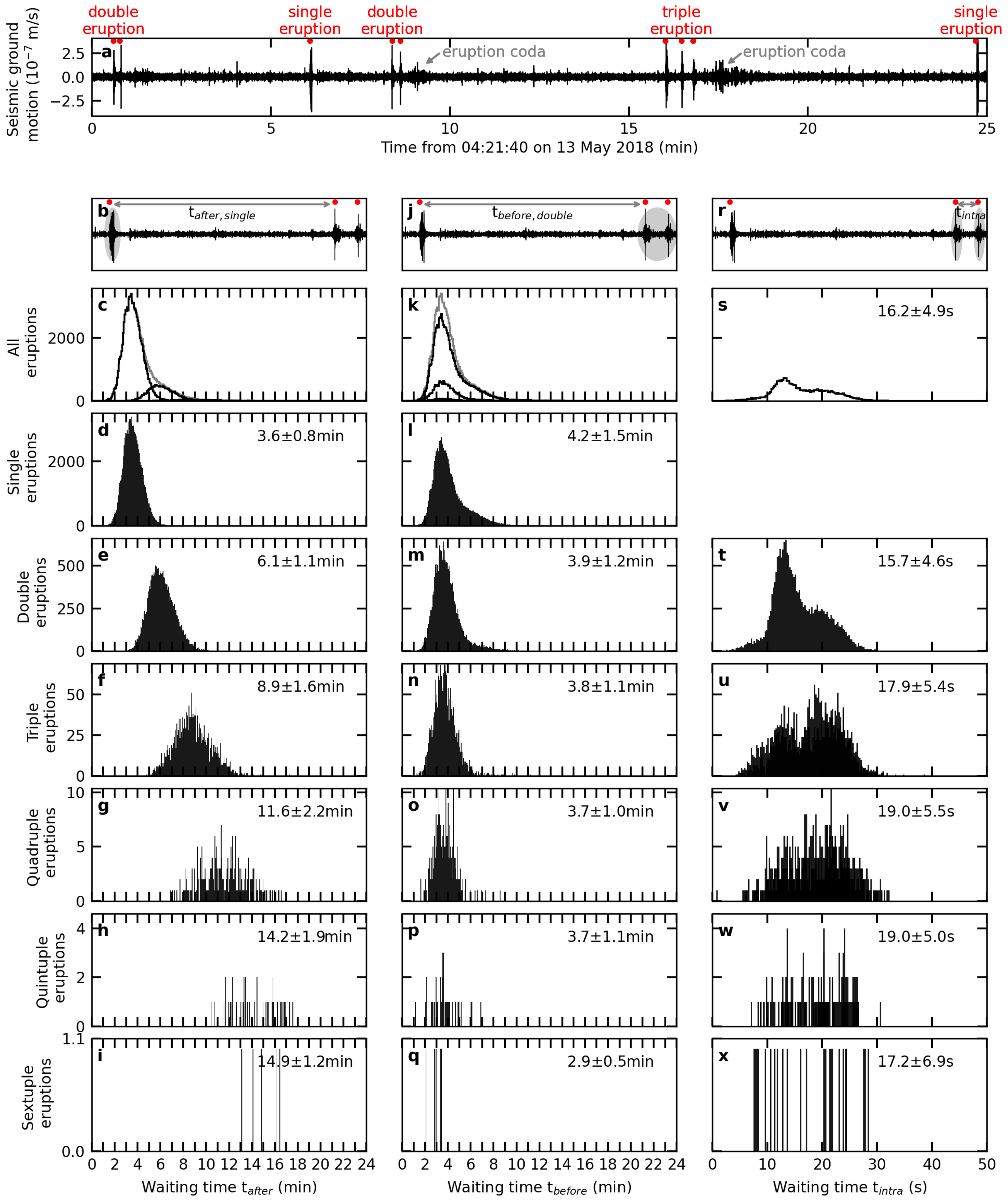

Figure S2. Eruption time statistics for 144 690 water fountains of Strokkur for eruptions between 28 June 2017 and 10 June 2018. The water fountains are separated into 97 162 single, 20 059 double, 2 077 triple, 238 quadruple, 39 quintuple and 5 sextuple eruptions following the classification scheme of Eibl[1] (a) Exemplary seismic waveform where different eruptions are marked in red. (b) Definition of time after eruption. (c-i) Time after eruption plotted following (c) all eruptions (grey lines) and for eruption types (black lines), (d) single eruptions, (e) double eruptions, (f) triple eruptions, (g) quadruple eruptions, (h) quintuple eruptions and (i) sextuple eruptions. (j) Definition of time before eruption. (k-q) Same as subfigures (c-i) where the times before eruption are sorted according to the following eruption type. (r) Definition of t_{intra}. (s-x) Same as subfigures (c-i) where t_{intra} is sorted according to eruption type.

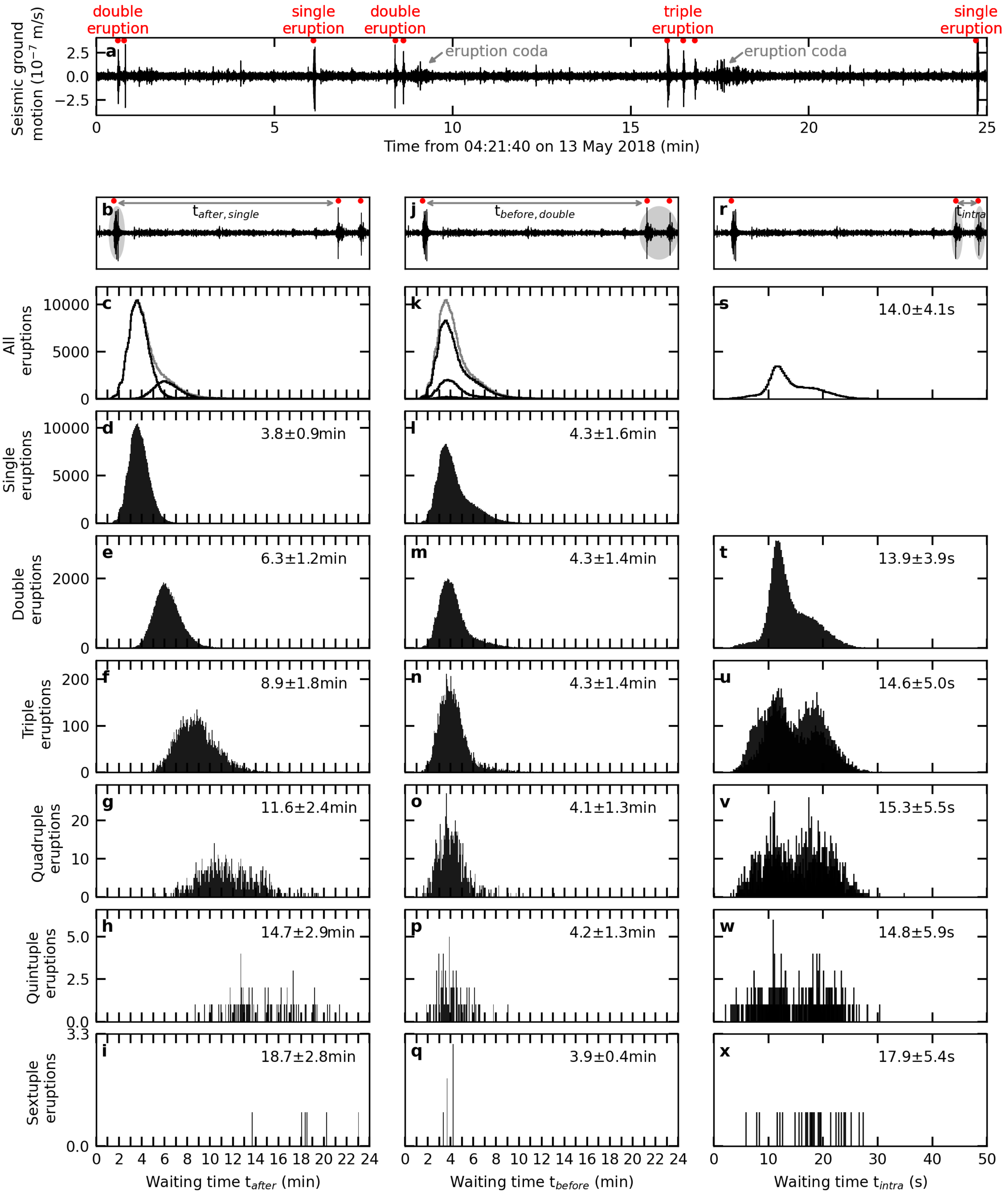

Figure S3. Eruption time statistics of Strokkur for 506 131 eruptions between 7 March 2020 and 30 September 2023. The water fountains are separated into 329 629 single, 75 627 double, 7 321 triple, 709 quadruple, 79 quintuple and 7 sextuple eruptions following the classification scheme of Eibl[1]. Subfigures as in Fig. S2.

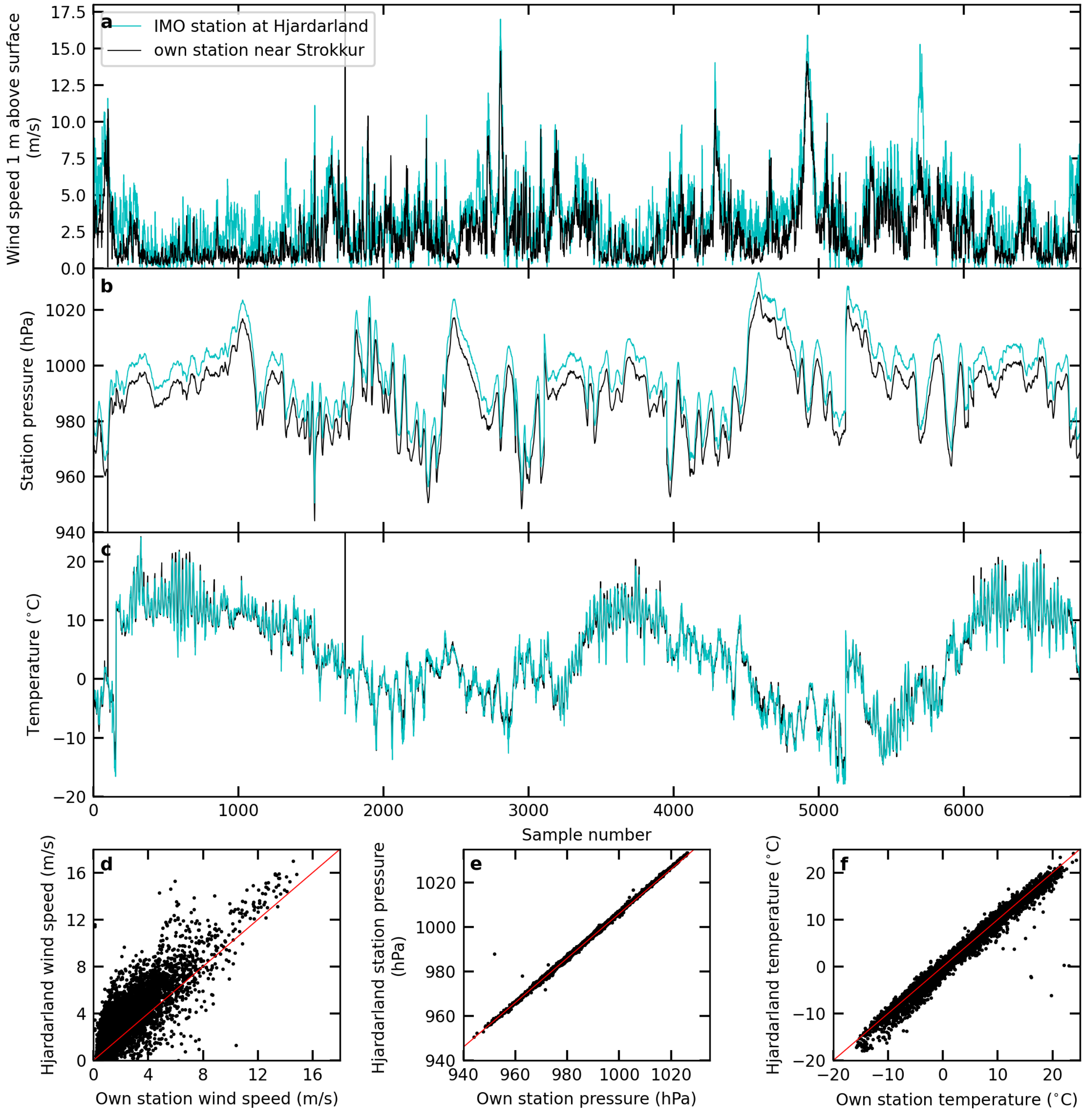

Figure S4. Comparison of our own weather sensor near Strokkur and the one at Hjarðarland (Fig. 1b) from March 2020 to April 2023. Due to data gaps at our own station at Strokkur, we show the time axis in samples and exclude times with no data. (a) Wind speed measured at 1 m height at Strokkur compared with the 10 m height measurement at Hjarðarland extrapolated to 1 m height according to the log wind profile $Ws_{z_2} = Ws_{z_1}/(log((z_2-d)/z_0)/log((z_1-d)/z_0))$[2] where z_2 is 1 m height, z_1 is 10 m height, Ws is the mean wind speed at the respective height. We assume a roughness length z_0 of 0.03 m for the roughness of open terrain (grassland) on wind flow and a zero-plane displacement d of 0 m where we assume the mean wind speed is zero[2]. (b) Comparison of station pressure and (c) temperature with time. (d-f) Direct comparison of 1 hour mean data measured by our own weather sensor and the one at Hjarðarland for the (d) wind speed, (e) station pressure and (f) temperature.

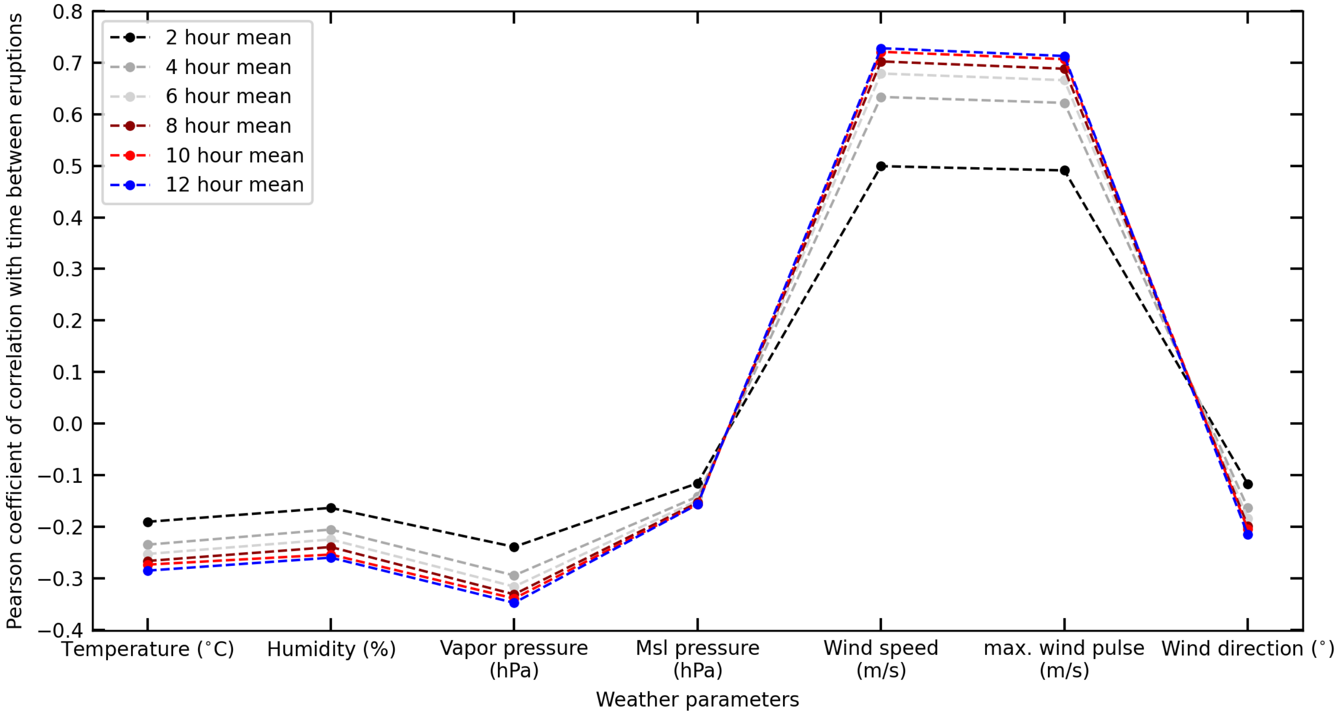

Figure S5. Binning weather parameters and recurrence intervals in 2 to 12 h long bins. A high correlation is seen for the wind speed. It increases with increasing window length but converges around 0.75. Temperature, humidity, vapor pressure, msl pressure and wind direction show no correlation with the recurrence intervals.

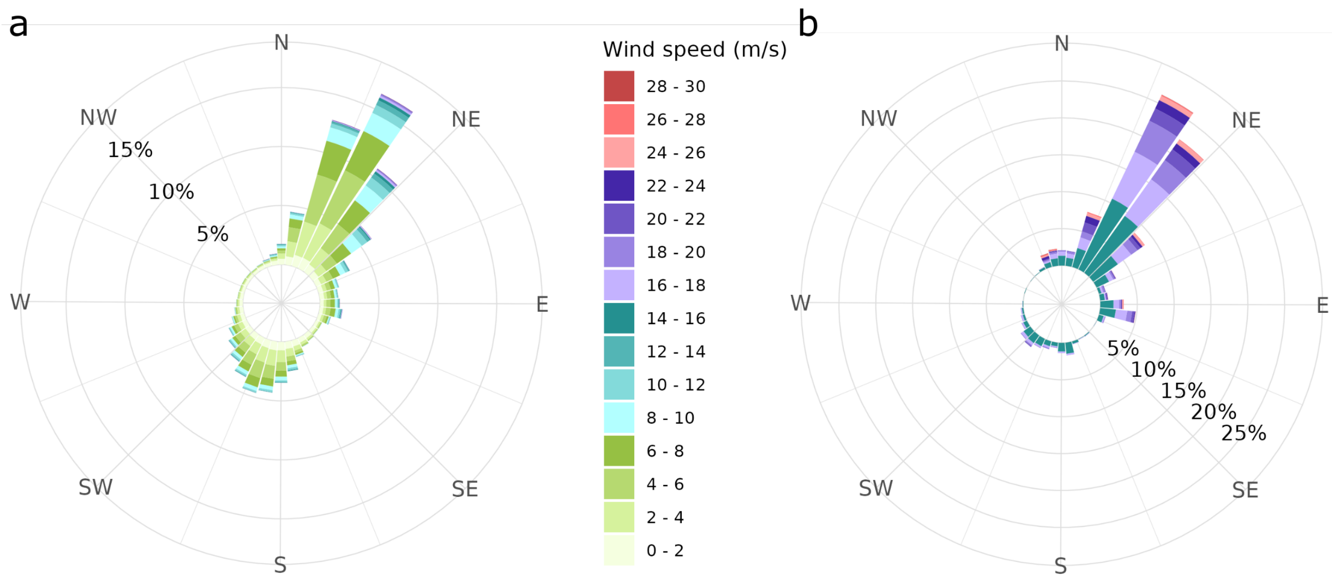

Figure S6. Windroses highlighting the dominant wind directions in 10° wide bins measured at 10 m height at Hjarðarland, Iceland, from 2017 to 2023 for 10 min data. The wind is blowing from the indicated direction where the length of the bins indicate the frequency and the colors the wind speed. (a) All wind speeds above 0 m/s. 3,9% of the data with 0 m/s wind speed were excluded from the plotting. (b) Only wind speeds of more than 14 m/s plotted.

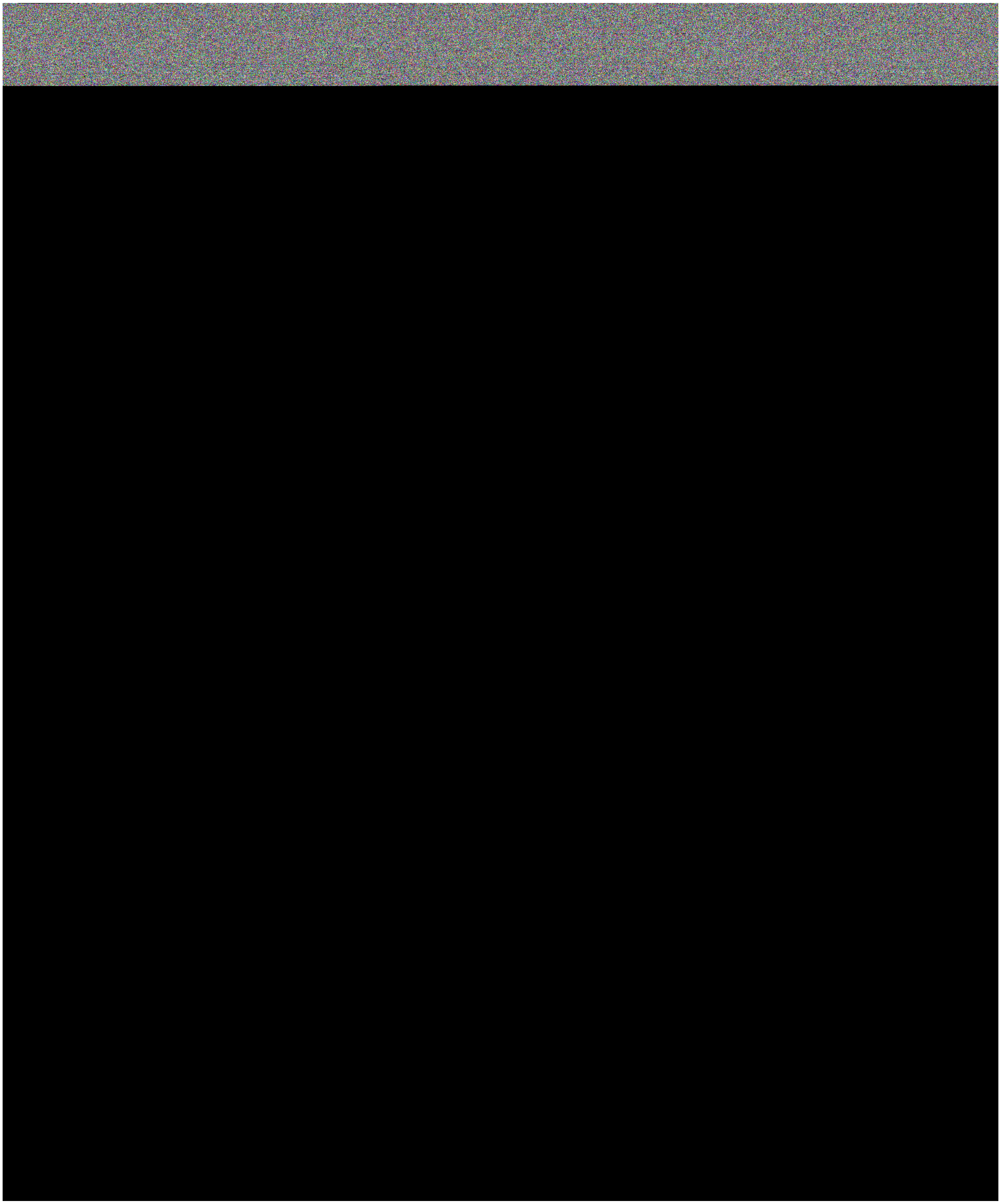

Figure S7. Overview of weather parameters and geyser eruptions from March 2020 to September 2023. (a) Msl pressure, (b) wind speed, (c) air temperature and (d) vapor pressure measured in 10-minute bins at Hjarðarland (black dots). (e) Number of water fountains and (f) mean recurrence interval of fountains per hour (black dots). Mean (red line) and standard deviation (gray line) in 12 hour bins in all subfigures.

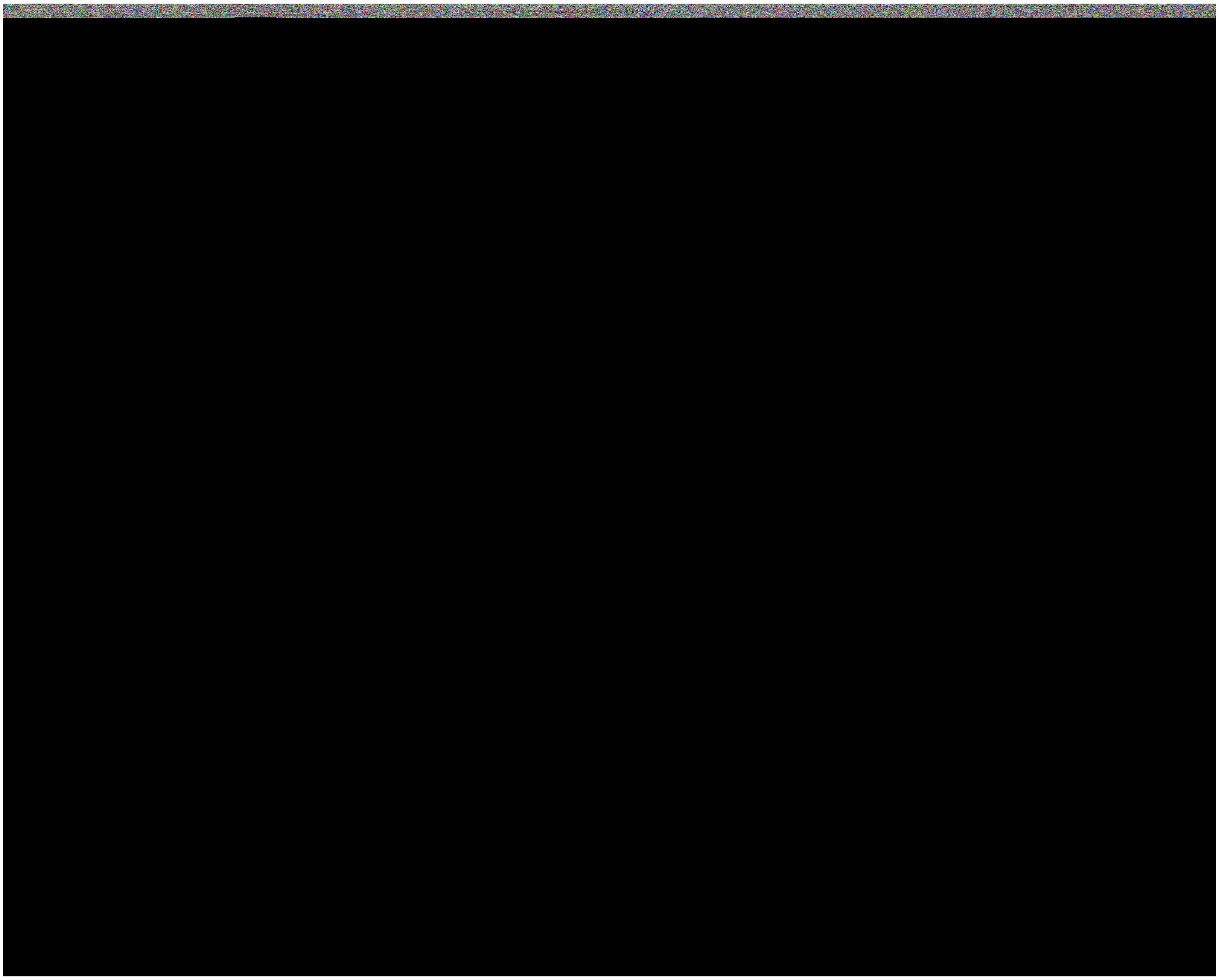

Figure S8. Zooming into three interesting time periods featuring large recurrence intervals. (a) Time between eruptions, (b) wind speed, (c) msl pressure, (d) humidity, (e) air temperature from 23 to 29 October 2020. (f-j) Same as subfigures (a-e) for 3 to 8 April 2020 and (k-o) 18 to 29 January 2021. The pronounced pressure drop and increased pressure gradient causes the the wind speeds to increase and are a characteristic pattern for a cyclone. In general, cyclonic activity dominates weather in Iceland during winter.
